# Potential roles for BACE inhibitors in AD therapeutic trials

**DOI:** 10.1002/alz.094708

**Published:** 2025-01-09

**Authors:** Paul S. Aisen

**Affiliations:** ^1^ Alzheimer’s Therapeutic Research Institute, Keck School of Medicine, University of Southern California, San Diego, CA USA

## Abstract

**Background:**

Now, at the beginning of the era of disease‐modifying drugs for AD, many questions must be addressed in the effort to optimize benefits. Important issues remain regarding the timing and duration of amyloid‐removing immunotherapy and the potential advantages of combination therapies. Anti‐fibrillar amyloid antibodies effectively remove amyloid but may not be appropriate for maintenance of the low amyloid state; secretase inhibition is a reasonable alternative. Evidence suggests that the earlier amyloid is removed the larger the benefit; by extension, beginning anti‐amyloid therapy before plaque accumulation may be optimal but may require targeting the generation of amyloid monomers. The toxicity of diffusible amyloid species suggests that combining immunotherapy with secretase inhibition may provide additive benefit. As reviewed in this session, the availability of substantial pre‐clinical and clinical data on BACEi therapy suggests that this approach can provide fine‐tuned reduction in amyloid peptide generation while avoiding the reversible cognitive toxicity associated with this class of drug.

**Methods:**

Findings from trials with amyloid‐removing antibodies and various BACE inhibitors along with growing data on accurate plasma markers of AD neurobiology inform considerations of the next generation of trials needed to clarify the role of BACEi therapy for disease‐modification.

**Results:**

Experience from ongoing clinical trials indicates that plasma biomarkers, particularly mass spectroscopy assays of abeta and ptau217 ratios, accurately predict amyloid levels by PET below the normal range. Further, changes in plasma ptau assays precede reaccumulation of fibrillar amyloid after cessation of amyloid‐lowering immunotherapy. These findings suggest that marrying accurate monitoring of plasma markers to BACEi therapy can provide a path to optimization of anti‐amyloid therapy. Future studies may demonstrate that screening individuals in late‐middle age with these plasma measures facilitates management of amyloid dysregulation at the pre‐plaque stage for primary prevention of AD.

**Conclusion:**

Coupled with plasma biomarkers of AD pathology, BACE inhibitor therapy may provide a feasible approach to the optimization of anti‐amyloid therapy for established AD as well as primary prevention of the disease.

